# Suburban Home School

**Published:** 1861-12

**Authors:** 


SUBURBAN HOME SCHOOL — under the superintendence and proprietorship of Rev. Alonzo Shears, M.A., Rector, who may be addressed at New-Haven, Connecticut, for circulars containing terms, references, etc., who receives into his family a limited number of boys, so as to make this a strictly family school, with ample play-grounds and all the conveniences for the preservation and promotion of the health of the students. We cordially commend this conscientiously conducted and favorably known institution to the patronage of our subscribers.

